# Bone Trabecular Pattern Analysis in Odontogenic Cysts Using Cone Beam Computed Tomography: A Clinical Retrospective Study

**DOI:** 10.7759/cureus.54452

**Published:** 2024-02-19

**Authors:** Induja Murugesan, Jayanth Kumar Vadivel, Karthikeyan Ramalingam

**Affiliations:** 1 Oral Medicine and Radiology, Saveetha Dental College and Hospitals, Saveetha Institute of Medical and Technical Sciences, Saveetha University, Chennai, IND; 2 Oral Pathology and Microbiology, Saveetha Dental College and Hospitals, Saveetha Institute of Medical and Technical Sciences, Saveetha University, Chennai, IND

**Keywords:** 3d dental imaging, dentigerous cyst, radicular cyst, odontogenic cysts, fractal

## Abstract

Introduction

The cysts of the maxillofacial region account for one of the most common pathologies of the head and neck region after the mucosal pathologies. Radiography provides an essential clue in early diagnosis and triaging, but it continues further as it is used to evaluate the post-treatment outcome. However, manual analysis is prone to errors. In this scenario, fractal analysis (FA) in radiographs uses mathematical methods to analyse the changes in grey scales in a given radiographic image. FA in odontogenic cysts is used to characterise their complexity, uncover hidden patterns, monitor treatment response, and potentially provide prognostic information. This paper aimed to assess the fractal characteristics of the radicular cyst (RC), dentigerous cyst (DC), and odontogenic keratocyst (OKC) using cone beam computed tomography (CBCT). The objective was to calculate fractal dimension (FD) values expressed in each of these cysts, which could prove to be a radiological adjunct in diagnosing the above cysts.

Materials and methods

As this is a retrospective study, the archives of CBCT images from June 2021 to December 2023 were obtained from patients diagnosed and confirmed with a histopathological diagnosis with RC, DC, and OKC. The FA was performed using Image J Software (Ver 1.51, National Institute of Health Bethesda, Fiji). The cortical and cancellous bones were segmented using thresholding techniques and converted to binary images. The mean FD of the three planes was then compared to establish the distinctive fractal characteristic for the specific odontogenic cysts. A one-way ANOVA was performed using the Statistical Product and Service Solutions (SPSS) (version 23.0; IBM SPSS Statistics for Windows, Armonk, NY) to determine the difference between FD values of RC, DC, and OKC with a significance level less than 0.05.

Results

The FD values of DC, RC, and OKC were 1.33 ± 0.17, 1.08 ± 0.16, and 1.65 ± 0.12, respectively. The results indicated that OKC had higher FD values than DC and RC, which means that OKC had lesser bone destruction compared to DC and RC. Inferential statistics showed that the one-way ANOVA was used to compare the means of the three groups of FD data. When calculated for the three groups, the F-statistic value was at 7.29, which yielded a P value of 0.03, making it statistically significant for a 95% confidence interval (p<0.05).

Conclusion

Our CBCT study on bone trabecular pattern analysis using FD and FA in odontogenic cysts reveals distinct alterations in bone parameters among different cyst types. The probability of higher FD values in OKC is because of lesser cortical bone destruction in OKC compared to the other cyst types. These findings have potential implications for diagnosing, treating, and prognosticating odontogenic cysts.

## Introduction

The cysts of the maxillofacial region account for one of the most common pathologies of the head and neck region after the mucosal pathologies. Among the cysts, the odontogenic cysts, those that have an odontogenic epithelial origin, are the most common, as indicated by a literature survey [1]. The above point suits adult and paediatric populations and both genders [1]. A cyst, as defined by Kramer (1974), is defined as a pathological cavity having fluid, semi-fluid, or gaseous contents, which is not created by the accumulation of pus with or without an epithelial lining [2]. An epithelial cyst is formed by the remnants left behind along the lines of fusion, which are triggered to proliferate and cause a cyst formation. Indeed, this accounts for the most common etiopathology for forming odontogenic cysts [2]. The radicular cyst (RC) is the most common of the odontogenic cysts, accounting for more than 60% [3]. The RC is most commonly seen in the fourth and fifth decades of life. Most cysts present as incidental findings on radiographs as they are clinically asymptomatic unless they become secondarily infected. In an observation by Mortensen et al., it was reported that cysts more than 15 mm tended to be symptomatic because of the tendency for perforation of the plates, leading to secondary infection [4]. The second most common cyst reported in the maxillofacial region was the dentigerous cyst (DC), which is caused by fluid accumulation between the developing crown and the reduced enamel epithelium. This cyst accounted for 20.6% of all odontogenic cysts in a study by Johnson et al. [5]. DC, being developmental cysts, tended to occur more frequently in the first and second decades of life, with the mandibular third molar being the most common site [6]. The next commonest cyst noted was the odontogenic keratocyst (OKC), formed from the cystic transformation of the dental lamina, also known as the cell rests of Serre [7]. OKC has a unique clinical behaviour with a recurrence potential, which prompted the WHO to classify it as a tumour in 2005 (as a keratocystic odontogenic tumour). However, the term was dropped by the WHO in 2017 and placed in the cyst category [8]. OKC was reported with a prevalence of 11% among all odontogenic cysts [9]. It has a peculiar clinical growth pattern that tends to grow among the central portion of the bone without causing buccolingual expansion. The cyst is commonly reported in women and is frequently seen in the first two decades of life [9].

Radiography provides an essential clue in early diagnosis and triaging, but it continues further as it is used to evaluate the post-treatment outcome. The different radiographs that were used include intraoral periapical radiographs, panoramic radiographs, computed tomography (CT), cone beam computed tomography (CBCT), and magnetic resonance imaging (MRI) [10]. The 2D imaging modalities have an inherent drawback of lack of visualisation in the buccolingual/palatal direction, and at least 30% mineral loss has to be present for it to be seen on a radiograph.

It is in this context that the 3D imaging modalities prove very worthwhile. Herein, CBCT offers the advantage of lower cost, reduced radiation, and excellent complex tissue resolution [11]. The above advantages prove handy in detecting the exact spread of the lesion. However, in CBCT exposures, unlike CT scans wherein we can derive the details of the internal structure of the lesion based on the Hounsfield units, it is impossible as the quantification of the radiodensity is based on the grayscale values [12]. Here, a finer analysis of the bone architecture in a quantitative rather than a qualitative manner can prove helpful.

The trabeculae of the cancellous bone resemble infinite shapes and sizes, which are mathematically complex. Here, the analysis of these shapes, scientifically referred to as fractals, provides insights into the diagnosis. Fractal analysis (FA) and fractal dimensions (FD) describe a ratio (index) of the complexity of these trabecular shapes as a non-integer. FA has been used to characterise the different cysts, detect early bone changes, monitor treatment responses, and provide potentially prognostic information [13].

The study aimed to analyse the fractal patterns of DCs, OKCs, and RCs using CBCT. The objective was to check for specific patterns expressed in each cyst, which could be a radiological adjunct in diagnosing the above cysts.

## Materials and methods

The Institutional Review Board of Saveetha Dental College approved this retrospective imaging study with the approval number IHEC/SDC/OMED-2105/23/228. The case sheet archives were searched from the Dental Information Archival System (DIAS) for diagnosing RC, DC, and OKC. The sample size was calculated for a power of 80 with a 95% confidence interval using prior literature. It was decided to include 15 samples under each category of RC, DC, and OKC [13].

The inclusion criteria for the study were as follows: cases with complete follow-up of radicular, dentigerous, and odontogenic keratocysts and all cases when presented with histopathological confirmation. The exclusion criteria for the study were as follows: CBCT images of cases that were distorted and cases where there was no follow-up.

This being a retrospective study, the archives of CBCT images from June 2021 to December 2023 of the cases fulfilling the criteria were retrieved from the Oral Medicine and Radiology department. The patients were scanned using CS 9600 (Carestream LLC, Atlanta, USA). The exposure parameters were based on the patient's age and build, ranging from 90-110 kilovoltage peak (KvP) to 8-10 milliamperage (mA). The CBCT images were collected in the native Digital Imaging for Communication in Medicine (DICOM) format. The primary image analysis in terms of the extent and size of the lesion in the orthogonal planes was obtained.

**Figure 1:**
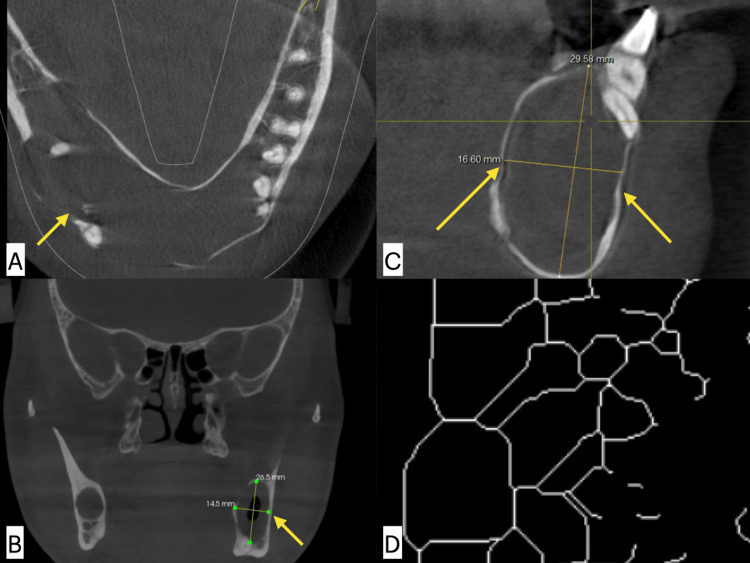
Odontogenic keratocyst (OKC) in CBCT images measurement of the size of the lesion in (A) axial, (B) coronal, (C) sagittal plane, and (D) final fractal image. CBCT: cone beam computed tomography

The collected images were imported into ImageJ software (ver 1.51, National Institute of Health Bethesda, Fiji). The software is freeware, and it can be downloaded online (https://imagej.nih.gov/ij/download.html). FA was utilised to determine the fractal dimensions at multiple points, with five points to mark a region being selected within each of the three orthogonal planes centred around the epicentre of the lesions. The mean fractal dimension value was calculated by the lesion boundary marked in the three planes. This gave the fractal characteristics for the specific cyst (Figure 1).

Fractal analysis (FA) procedure

**Figure 2:**
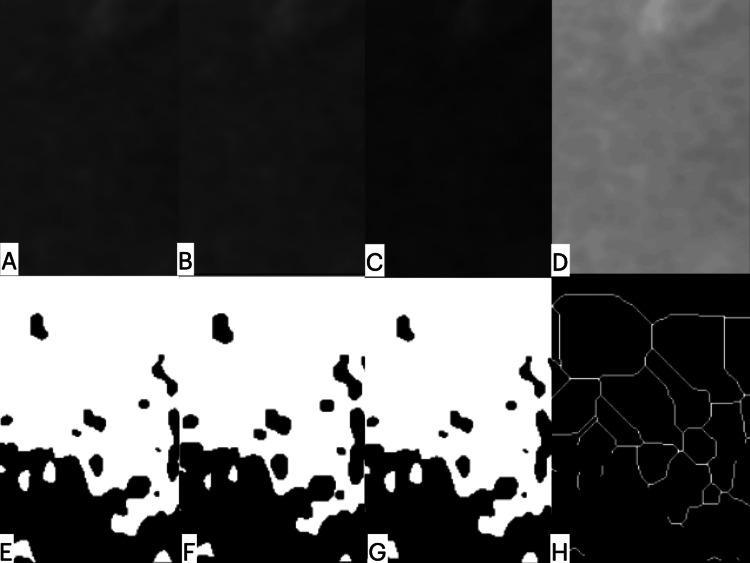
Fractal analysis: step by step. The images converted to 8-bit images (A). This image was duplicated and blurred by Gaussian filter (B). The resulting image was blurred and subtracted with a background image (C). The value of 128 was added (D). The image transformed to binary thresholding (E). The resultant image was eroded and diluted (F,G). The image was skeletonised (H).

All procedures for calculating the FD were performed using Image J software (ver 1.51, National Institute of Health Bethesda, Fiji). Image J software was used to analyse the images and possibilities for changes in the region of interest (ROI) trabecular pattern as 60 x 60-pixel-sized squares. A standard method from the literature was used [14]. The following steps were followed: 1) ROI image duplication; 2) image blurring (from Gaussian) with 33 radii (pixels), which removes all the fine and medium scale structure and retains only significant variations in density (low-pass filter); 3) image subtraction, wherein the blurred image was subtracted from the original of the same patient; 4) adding, wherein a constant value is added to each pixel of the result subtracted image (this generates an image with an average value of about 128, regardless of the original image intensity, discarding gross variations in the intensity); 5) binary transformation, wherein the previous image was transformed into a binary image that is black and white; 6) erode, wherein the last result was “eroded”; 7) dilate, wherein the previous result was “dilated,” with each pixel loaded with a maximum value in the vicinity of 3x3; and 8) skeletonized, wherein the edges of the pixels of the binary-object-dilated-eroded-inverted images were removed until they were ducted to a single vast skeleton of a single pixel. The object (trabecular) is black, and the background is white. The entire process is outlined in the image series generated from our image analysis (Figure 2).

**Figure 3:**
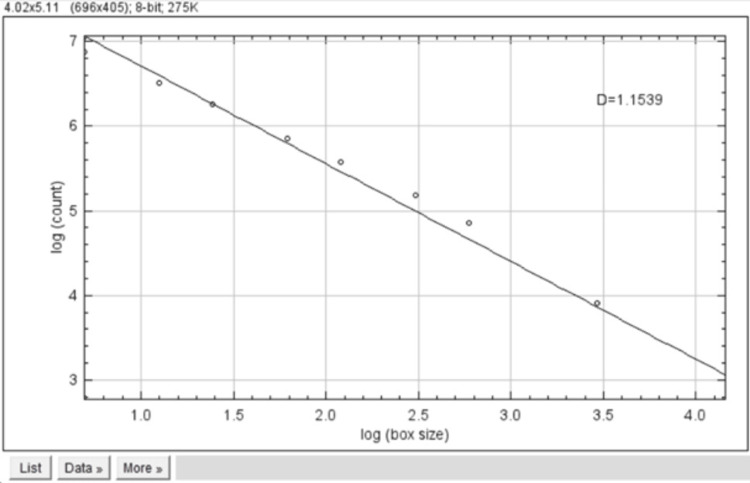
Fractal dimensions of skeletonised images were calculated using fractal box count.

The skeletonised images were calculated using the fractal box count method, which included box values (2, 3, 4, 6, 8, 12, 16, 32, and 64). A graph was obtained between box count and box size in log values, which showed the FD values. This analysis provides us with the value of FD (Figure 3).

**Figure 4:**
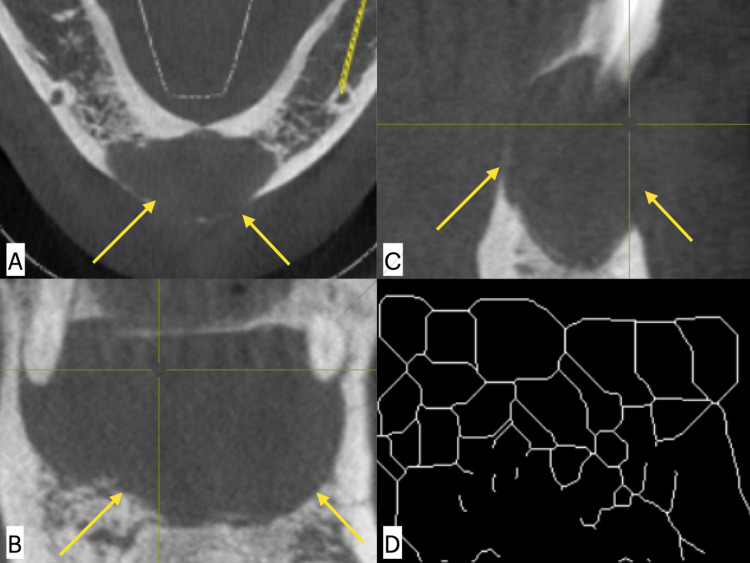
Radicular cyst (RC) in CBCT images measurement of the size of the lesion in (A) axial, (B) coronal, (C) sagittal planes, and (D) skeletonised fractal image. CBCT: cone beam computed tomography

In the same manner, this was done for the RC. The presence of an RC is diagnosed by a periapical radiolucency about a nonvital tooth, and Figure 4 shows a representative image of RC.

**Figure 5:**
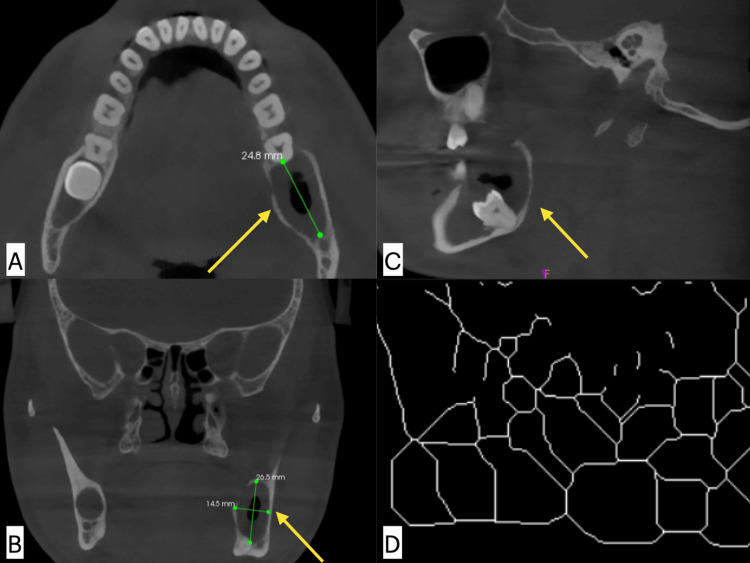
Dentigerous cyst (DC) in CBCT images measurement of the size of the lesion in (A) axial, (B) coronal, (C) sagittal planes, and (D) skeletonised fractal image. CBCT: cone beam computed tomography

The image below represents the DC, identified by a perifollicular radiolucency surrounding the tooth's crown (Figure 5).

Descriptive and inferential statistics

The Statistical Product and Service Solutions (SPSS) (version 23.0; IBM SPSS Statistics for Windows, Armonk, NY), a statistical software for social sciences, was used for statistical analysis. At the initial level, descriptive statistics were performed to analyse the age and gender distribution of the patients. At the next level, inferential statistics was conducted using a one-way ANOVA to compare FD values, with the statistical level of significance being fixed at less than or equal to a P value of 0.05.

## Results

**Figure 6:**
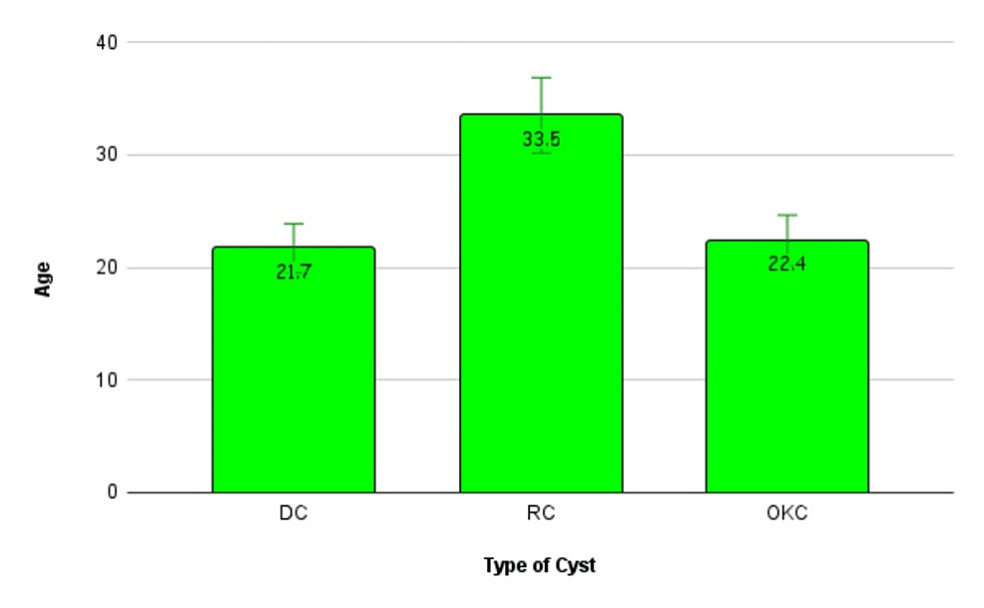
The bar graph distribution of the ages of the patients included in the study. DC: dentigerous cyst RC: radicular cyst OKC: odontogenic keratocyst

There were 15 cases in each of the three cyst categories. The mean age with the standard deviations is given. The bar graph representation of the age of the cysts with the error bars is given (Figure 6).

The gender distribution of the samples selected for the analysis is given (Table 1).

**Table 1:** Gender distribution of the samples.

Name of the cyst	Males	Females
Dentigerous cyst	10	5
Radicular cyst	7	8
Odontogenic keratocyst	6	9

**Figure 7:**
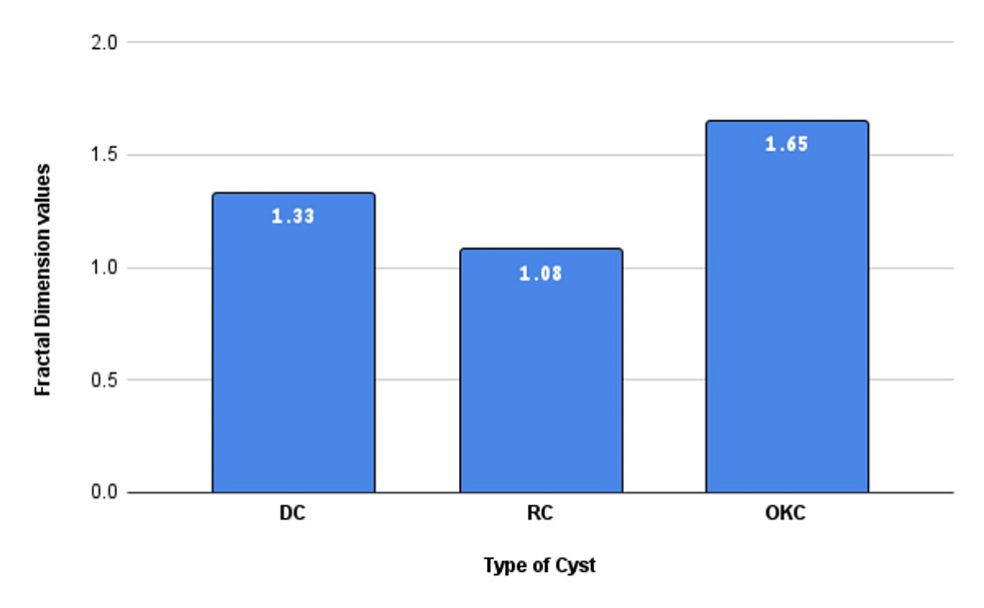
Graph showing that the OKC had higher fractal dimension values than DCs and RCs. DC: Dentigerous cyst RC: Radicular cyst OKC: Odontogenic keratocyst

The graphical presentation of the fractal values is given in the bar graph below (Figure 7).

The comparative mean FD values across the three cyst groups are given (Table 2).

**Table 2:** Mean fractal values of the three cyst groups.

S.NO	Cyst	Mean ± SD
1	Dentigerous cyst	1.33 ± 0.17
2	Radicular cyst	1.08 ± 0.16
3	Odontogenic keratocyst	1.65 ± 0.12

As the next part of the descriptive analysis, we studied the FD values for each cyst category to either gender or location of the lesional site. The comparison is given (Table 3).

**Table 3:** Average fractal dimension values between the two gender groups in DC, RC, and OKC.

Cyst	Gender	Mean ± SD	P value
Dentigerous cyst	Male	1.11 ± 0.18	P=0.13
	Female	1.15 ± 0.16	
Radicular cyst	Male	1.12 ± 0.09	P=0.32
	Female	1.03 ± 0.25	
Odontogenic keratocyst	Male	1.6 ± 0.15	P=0.23
	Female	1.73 ± 0.03	

Inferential statistics

The age of the patients was found to follow the normal distribution of data as per Shapiro-Wilk’s test, and parametric tests were used. Hence, a one-way ANOVA was used to compare the means of the three groups of FD data. When calculated for the three groups, the F-statistic value was at 7.29, which yielded a P value of 0.03, making it statistically significant for a 95% confidence interval (p<0.05). As the next step, a post-hoc analysis was done using Tukey’s Honest Significant Difference (HSD) test to analyse the means of the groups that yielded the values (Table 4).

**Table 4:** Comparison of fractal dimension values between the subgroups.

Group compared	Q value	P value
Odontogenic keratocyst cyst vs. dentigerous cyst	4.08	0.03
Odontogenic keratocyst cyst vs. radicular cyst	0.24	0.01
Dentigerous cyst vs. radicular cyst	1.03	0.75

The above data show that the data comparison between OKC vs. DC and RC was statistically significant. Moreover, we compared the FD values in each category of cysts between the genders using the t-test. The P value, when compared between the genders, did not reveal statistically significant p-values (Tables 3-4).

## Discussion

The cysts of the jaw are categorised into odontogenic and non-odontogenic based on their epithelial origin. Further, based on their aetiology, they are classified as inflammatory and developmental. The three most common cysts reported in the maxillofacial region are RC, DC, and OKC [1,2]. The mean age of the two developmental cysts, viz., the DC and OKC, was 21.7 and 22.4, respectively. This is in line with what is reported in the literature, as both cysts are seen in the second to third decade of life [2]. However, looking at the gender distribution, RC and OKC were seen predominantly in the females, but DC was seen commonly in the males. However, the sample size was too small to make any decisive conclusion. A literature survey also shows that the cysts are predominantly seen in women [1,2]. With the analysis of the demographics, the following parameter was taken up to compare the FD values. FA is a non-traditional mathematical method to decipher the metric values behind complex irregular shapes such as the trabeculae dimensions of the cancellous bone. Herein, the shapes are converted into closer geometric shapes using software. In the next stage, the dimensions of the geometric shapes are calculated and mentioned as FD [13]. The subtle alterations of the core bone in pathological alterations can easily be detected using FD, and the entire process can also be automated.

The role of radiology herein is to report on the exact anatomical extent of the cysts and provide more detailed information on the location of the cysts. Most of the time, with 2D imaging, the same anatomical spread of the lesion becomes challenging to diagnose because of anatomic superimpositions. In this scenario, using 3D imaging modalities such as CBCT provides us an advantage in diagnosing. The complex information provided by CBCT can be deciphered and broken down by FA, and the FD values calculated makes us make a diagnosis easier. Further, using automated tools also gives way to employing artificial intelligence in creating a diagnosis [13]. Cancellous bone presents a complex interplay of trabecular patterns, which can be broken down into definite geometrical shapes, making mathematical fractal pattern analysis possible. FA and FD analysis have been used predominantly to quantify the bone pattern changes in temporomandibular joint pathologies, osteomyelitis, and bone regeneration [14]. Moreover, they have been used in periapical pathologies to differentiate between cysts, abscesses, and granulomas [13]. Thus, in this paper, we analysed the FD values of three cysts.

RC represents the most common odontogenic cyst of an inflammatory origin arising from Malassez cell rests [4]. In a prior study done by Saeed et al., it was seen that the pixel intensity of the internal aspect showed that the cysts had the least pixel intensity compared to granuloma, which is acceptable because of the presence of fluid content within the cyst compared to the presence of solid tissue within the lesion [15]. In the same study, the FA values for a cyst were 1.26, which is comparable to the result obtained in our study. However, their sample size was only 6, and they used panoramic radiography, while we used CBCT. In another follow-up study by Lim et al. of the assessment healing of RC, FA analysis was conducted using panoramic radiographs. The preoperative FA values were 1.10, much closer to the value obtained in our paper [16]. Hence, the FD values that are primarily determined by the amount of bone destruction within the cysts obtained in our study align with those obtained from the studies based on 2D imaging.

There are no prior studies of using FA in DC. In a case report by Astuti et al., it was reported that the pretreatment FD values in OKC were 1.46 and, after marsupialisation, rose to 1.57 post treatment after 11 months [17]. The values calculated for OKC in our paper also align with those obtained. However, studies have not been done with OKC on a larger sample.

FA has been extensively used in medicine and is now being explored in dental specialties. It is primarily used in dental imaging to analyse bone patterns. It has been used to assess trabecular patterns in cases of osteoporosis. This is a more accessible and standardised method to evaluate bone mineral density [18,19]. The usage of FA on panoramic radiographs has been extensively reported, but only some studies are based on CBCT. The advantage of high-resolution images in CBCT is that they allow us to evaluate bone quality accurately. Prior studies have shown that comparing panoramic radiographs and CBCT has yielded more accurate FA values comparable to bone mineral density assessment using dual-energy X-ray absorptiometry [20,21].

In cases of cysts or osteolytic lesions, there has been a decrease in FD values, observed in the study by Heo et al. in the survey of subchondral cysts in temporomandibular joint disorders [22]. The role of FA and FD in the diagnosis of cysts proves helpful. It can be an adjunct to histological analysis, especially when it presents similar clinical presentations, such as the association of an impacted tooth and OKC. Analysing the trabecular pattern would be a supplementary tool in those aspects. Further, it can assess the follow-up of bone regeneration in case of cysts associated with an inflammatory aetiology [14].

From this study, it is evident that such analysis of the trabecular bone will help detect cysts, which could be integrated into artificial intelligence and machine learning in the radio diagnosis of cysts. Further, these could also be used in patients who are medically compromised, wherein there are contraindications for biopsy procedures [23]. Such radiographic procedures can be utilised instead of incisional biopsies to arrive at a diagnosis.

The study's main limitation was unicentric data from a single ethnic group. The study did not evaluate the impact of CBCT exposure parameters such as field of view, KvP, and voxel size on the FD values. Future studies incorporating the above confounding factors would be needed to standardise the values. Moreover, it is helpful to explore the possibility of longitudinal studies with follow-ups to help understand the trabecular remodelling occurring in the cysts after the treatment.

## Conclusions

In conclusion, our CBCT study on bone trabecular pattern analysis using FD and FA in odontogenic cysts reveals distinct alterations in trabecular parameters among different cyst types. These findings have potential implications for diagnosing, treating, and prognosticating odontogenic cysts. With further research and clinical validation, trabecular pattern analysis could become integral to the comprehensive assessment of odontogenic cysts, contributing to improved patient care and oral and maxillofacial pathology outcomes. The data from the study give valuable insights into the usage of this tool as a supplementary diagnostic aid.
